# Natural Killer Cell Function, an Important Target for Infection and Tumor Protection, Is Impaired in Type 2 Diabetes

**DOI:** 10.1371/journal.pone.0062418

**Published:** 2013-04-25

**Authors:** Jeannig Berrou, Sophie Fougeray, Marion Venot, Victor Chardiny, Jean-François Gautier, Nicolas Dulphy, Antoine Toubert, Marie-Noëlle Peraldi

**Affiliations:** 1 Institut National de la Santé et de la Recherche Médicale (INSERM), UMR 940, Paris, France; 2 Assistance Publique-Hôpitaux de Paris (AP-HP), Hôpital Saint-Louis Centre d’Investigations Biomédicales “H-O-G”, Paris, France; 3 INSERM U775, Centre Universitaire des Saints Pères, Paris, France; 4 Université Paris Descartes, Sorbonne Paris Cité, Paris, France; 5 AP-HP, Hôpital Saint-Louis, Department of Diabetes and Endocrinology, Paris, France; 6 Université Paris 7-Denis Diderot, Sorbonne Paris Cité, Paris, France; 7 Université Paris Diderot, Sorbonne Paris Cité, Institut Universitaire d’Hématologie, Paris, France; 8 AP-HP, Hôpital Saint-Louis, Nephrology and Transplantation Department Paris, France; University of Sydney, Australia

## Abstract

Patients with Type 2 diabetes (T2D) are highly susceptible to infection and have an increased incidence of some tumors, possibly due to immune system dysfunction. In the innate cellular immune system, Natural Killer (NK) lymphocytes are important effectors responsible for controlling infections and combating tumor development. We analyzed NK cell subsets in 51 patients with long-standing T2D. Compared with healthy blood donors, diabetic patients showed a profound decrease in both NKG2D-positive NK cells (44% *vs.* 55.5%, P<0.01) and NKp46-positive cells (26% *vs.* 50%, P<0.01). Decreased expression of these receptors was associated with functional defects, such as reduced NK degranulation capacity when challenged with the tumor target cell line K562 (10.3 *vs.* 15.8%, P<0.05). This defect could be restored *in vitro* by stimulating NK cells from T2D patients with IL-15 (P<0.05). NKG2D expression was found to be negatively correlated with HBA1c level (r = −0.50; P = 0.009), suggesting that sustained hyperglycemia could directly influence NK cell defects. We demonstrated that endoplasmic reticulum (ER) stress, an important mediator in diabetes-associated complications, was inducible *in vitro* in normal NK cells and that tunicamycin treatment resulted in a significant decrease in NKG2D expression (P<0.05). Furthermore, markers of the Unfolded Protein Response (UPR) BiP, PDI and sXBP1 mRNAs were significantly increased in NK cells from T2D patients (P<0.05, P<0.01, P<0.05, respectively), indicating that ER stress is activated in vivo through both PERK and IRE1 sensors. These results demonstate for the first time defects in NK cell-activating receptors NKG2D and NKp46 in T2D patients, and implicate the UPR pathway as a potential mechanism. These defects may contribute to susceptibility to infections and malignancies and could be targetted therapeutically.

## Introduction

Clinicians are well aware that patients with type 2 diabetes (T2D) are highly susceptible to infections and are prone to malignancy [1], [2]. Although this predisposition has been known for decades, the underlying mechanisms causing this immune dysfunction remain unclear. With the emerging pandemics of diabetes and obesity, it is becoming even more important and urgent to identify the parameters associated with infection and malignancy in this context [3]. A limited number of recent studies have focused on immune dysfunction associated with hyperglycemia *per se*. For instance, acute hyperglycemia has been shown to reduce neutrophil degranulation in healthy subjects [4] and to down-regulates the LPS-induced activation pathway, as well as interleukin-1α production [5]. In diabetic patients, polymorphonuclear leukocytes have been shown to produce increased levels of reactive oxygen species, possibly as a result of the effects of hyperglycemia [6]. Oxidative stress was recently shown to modulate natural killer (NK) cell functions in a cohort of patients with end-stage renal failure who also show increased production of reactive oxygen species [7]. Thus, oxidative stress and endoplasmic reticulum (ER) stress induced by high glucose levels [8] may influence NK cell function in T2D patients. Metabolic syndrome has also been linked to a modified NK cell profile. In a recent study, Lynch et al. showed that obese non-diabetic patients had significantly fewer circulating NK and cytotoxic T lymphocytes (CTL) than matched lean controls [9]. The NK population is of particular interest in the context of T2D as NK cells are known to play an important role in defending the body against infections and tumors [10], [11].

NK cells have the machinery to recognize and deal with both tumors and viral or bacterial infections, all of which are prevalent in T2D. Their activity is regulated by activating receptors, including NKp30, NKp44, NKp46, NKG2C and NKG2D, which all bind ligands present at the surface of tumors or infected cells [12], [13]. Among these activating receptors, NKG2D is expressed on NK cells, CD8-positive TCRαβ T cells, and CD8-positive TCRγδT cells. One of the main NKG2D ligands, the MHC class I-related molecule MICA is expressed in endothelial cells, epithelial cells and monocytes [14], [15]. In these cells, surface MICA expression can be induced by various types of stress, such as genotoxic stress in tumors, infections (e.g. by *Mycobacterium tuberculosis*, or *Escherichia coli*) and oxidative stress [16], [17]. Another of the activating receptors, NKp46 was discovered through its recognition of cancer cells [18]. Activation of this receptor plays a major role in tumoral immunity and in host control of viral infection [19], [20]. Despite the potential involvement of NK cells in the secondary diseases related to T2D, NK cell receptor expression has yet to be specifically studied in samples from patients with T2D.

Hyperglycemia is prominent with T2D and has been shown to impair ER functions, leading to the accumulation of misfolded proteins in the ER lumen and promoting ER stress [21], [22], [23]. Recent experimental evidence suggests that ER stress in diabetic endothelial cells is an important contributor to diabetes-related vascular complications [24]. When ER stress occurs in such cells, it triggers a signal transduction pathway, the Unfolded Protein Response (UPR). The UPR limits the accumulation of unfolded proteins in the ER and helps to re-establish normal ER function. Three major sensors are involved in UPR signaling: PERK (Protein kinase RNA(PKR)-like Endoplasmic Reticulum Kinase), IRE1 (Inositol Requiring Enzyme 1) and ATF6 (Activating Transcription Factor 6). PERK activation reduces protein translation and therefore limits the accumulation of proteins in the ER. IRE1 activation (resulting in the alternative splicing of X-box binding protein XBP1 mRNA transcript) and ATF6 activation both promote the expression of downstream UPR target genes, including those coding for ER chaperones such as BiP (Binding immunoglobulin Protein) and PDI (Protein Disulfide Isomerase). Hyperglycemia can cause ER stress in β-pancreatic cells [25], but no data exist relating a similar effect in white blood cells including NK cells in T2D patients. In addition, it is currently unknown whether the UPR correlates with a decreased expression of some NK receptors.

The aim of the current work was to establish the potential role of NK cell abnormalities in the immune dysfunction observed in T2D patients. Our data show for the first time that T2D patients have a decreased frequency of both NKp46 and NKG2D-positive NK cells. Down-regulation of NKG2D on NK cells was also more pronounced in patients with uncontrolled hyperglycemia and appears to be linked to ER stress.

## Patients and Methods

### Study Population

Type 2 diabetic patients included in the study were regularly followed as out-patients in the Endocrinology Unit in our hospital. Inclusion criteria were as follows: patients older than 18 years with a diagnosis of T2D. Exclusion criteria included a history of malignancy, chronic or acute hepatitis B or C, or HIV infection, previous or current immunosuppressive treatments and current infection. Clinical data were obtained from medical files. Renal function was estimated by the MDRD equation [26]. The study included 51 patients with T2D. Blood samples from 54 age-matched healthy donors were obtained from the Transfusion Center at the Saint-Louis Hospital, Paris, France. This study was approved by the Institutional Review Board of Saint Louis Hospital, Paris, France, and all patients gave written informed consent for participation.

### Antibodies and FACS

Lymphocyte immunophenotyping was performed on frozen samples of PBMCs by eight-color analysis using a flow cytometer (Canto II) from BD Biosciences (Becton Dickinson, San Jose, CA, USA). Samples were fixed with PBS containing 2% paraformaldehyde. The following antibodies were used for the analysis: fluorescein isothiocyanate (FITC)-conjugated anti-KIR2DL2 (CD158b, clone CH-L); phycoerythrin (PE)-conjugated anti-NKp30 (clone Z25), anti-NKp44 (clone Z231), anti-NKp46 (clone BAB281), anti-NKG2C (clone 134591), anti-KIR2DL1DS1 (CD158a/h, clone 11PB6) and anti-KIR2DS4 (CD158i, clone FES172); peridinin chlorophyll protein (PerCP)-conjugated anti-CD8 (clone SK1); allophycocyanin (APC)-conjugated anti-NKG2D (clone ON72), anti-NKG2A (clone Z199), and anti-KIR3DL1 (CD158e, clone DX9); biotin-conjugated anti-CD56 (clone B159) used with streptavidin-PE-Alexa Fluor 750; allophycocyanin-H7 (APC-H7)-conjugated anti-CD16 (clone 3G8), AmCyan-conjugated anti-CD3 (clone SK7) and Pacific Blue-conjugated anti-CD4 (clone RPA-T4).

Products were purchased from Beckman Coulter (Miami, FL, USA), Becton Dickinson Biosciences (San Jose, CA, USA), R&D Systems Inc (Minneapolis, MN, USA). All flow cytometry analyses were performed using a combination of CD3, CD16, and CD56 antibodies along with a fourth antibody to specifically gate either CD3-positive T lymphocytes or the CD3-negative CD56^bright^ CD16^dim/neg^ and CD3-negative CD56^dim^ CD16^bright^ NK populations. The frequency of positive cells and the level of expression of the fourth marker in each subset were evaluated using the DIVA software (BD Biosciences, San Jose, CA, USA). To monitor the induction of cell death, NK cells were stained with fluorescein isothiocyanate-conjugated Annexin V (Becton Dickinson, San Jose, CA, USA) according to the manufacturer’s instructions.

The correspondance between colors and specificities is shown in supplemental data. (Table S1).

### PBMC and NK Cell Isolation

PBMCs from diabetic patients and healthy donors were isolated from 15 ml of fresh whole blood by density gradient centrifugation using lymphocyte separation medium (Eurobio, Les Ulis, France). PBMCs were stored in liquid nitrogen prior to phenotypic and functional analysis. When necessary, NK cells were then isolated from PBMCs by negative selection using an NK Cell Isolation Kit II (Miltenyi Biotec, Auburn, CA, USA) according to the manufacturer’s instructions.

### Cell Culture

Frozen PBMCs and isolated NK cells were thawed in RPMI 1640 containing 10% FBS. Where indicated, IL15 (R&D Systems, Lille, France) was added to NK cell cultures for 16 hours. Cells were harvested and stained with appropriate antibodies for flow cytometry analysis. PBMCs from healthy donors were cultured with tunicamycin (1.25 µg/mL; Sigma-Aldrich, Saint Quentin Fallavier, France) for 6 hours where indicated. Cells were harvested and stained with appropriate antibodies for flow cytometry analysis, or were lyzed in TRI-reagent (Euromedex, Souffelmeyersheim, France).

The K562 cell line, used as a target in degranulation assays, was maintained in RPMI 1640 containing 10% FBS.

### CD107a Degranulation Assay

A CD107a degranulation assay was used to assess functional activity of NK cells, as described [27]. Briefly, K562 target cells were incubated with PBMCs at an E:T ratio of 5∶1 for 16 hours at 37°C in RPMI 1640 containing 10% FBS and CD107a-PE (Becton Dickinson, San Jose, CA, USA). Thereafter, cells were incubated with CD56-Alexa Fluor 488 (clone B159), CD3-PerCP (clone SK7), NKG2D-APC (clone ON72) and CD16-APC-H7 (clone 3G8) antibodies for 20 minutes on ice. Cells were fixed in PBS-2% paraformaldehyde prior to flow cytometry analysis. The percentage of CD107a is the result of subtracting the spontaneous NK cells degranulation to the NK cells degranulation with K562 target cells.

### Real Time PCR Quantification

Depending on the experiment design, either purified NK cells or PBMCs were used for Real Time quantitative PCR assays. PBMCs were thawed and cultured in RPMI 1640 containing 10% FBS for 6 hours with or without tunicamycin (1.25 µg/mL). Cells were lysed in TRI-reagent (Euromedex, Souffelmeyersheim, France), and total RNA was isolated according to the manufacturer’s instructions. Reverse Transcription was performed using the Superscript III First-Strand Synthesis System for RT-PCR (Invitrogen, Cergy Pontoise, France). NKG2D, NKp46, ATF4, BiP, CHOP, GADD34, GRP94, HERP, PDI, sXBP1 mRNAs, as well as the reference GAPDH and RPL13A mRNAs were assayed using fluorescence-based real time PCR. Real-time PCR was performed on an ABI PRISM 7000 sequence Detection System with SYBR Green reagents (Applied Biosystems, Foster City, CA). After initial denaturation (95°C for 10 min), 40 cycles of a two-step PCR (15 s at 95°C and 1 min at 60°C) were performed. Relative expression was calculated based on the ΔΔC_T_ method with GAPDH and RPL13A as reference genes and cloned PCR products as calibrators. The primer sequences are listed in Table S2.

### Serum Preparation ELISA

Serum was obtained by centrifuging blood samples at room temperature for 15 min at 300×*g*. The resulting serum samples were transferred to polypropylene microtubes and stored at –80°C prior to analysis. Samples were thawed at room temperature before assaying serum levels of sMICA using commercial MICA enzyme-linked immunosorbent assay kits (Bamomab, Enzolife Sciences, Villeurbanne, France), and serum circulating IL15 was determined using commercially available IL15 enzyme-linked immunosorbent assay kits (R&D systems Inc, Minneapolis, MN USA). The assay was performed according to the manufacturer’s instructions and as previously described [7]. All samples were tested in duplicates.

### Statistical Analysis

Results are shown as the median value (range). Data were tested for inter-group statistical significance using Wilcoxon’s test for paired variables, or the Mann-Whitney U test for unpaired variables. Correlations were assessed using Spearman’s correlation coefficient. All tests were two-sided, and P<0.05 was considered as the threshold for a significant difference.

## Results

### Demographic Characteristics of the Population

The study population comprised 51 T2D patients and 54 age-matched healthy donors. The demographic breakdown for the diabetic patients is shown in Table 1. Blood samples for immunophenotyping, HbA1c levels, lipid profile, serum creatinin, and urine collection for microalbuminuria were collected on the same day. All the patients included had high HbA1c levels, indicative of some periods of hyperglycemia over the months preceding this study. Almost half the study population had a history of infection, having required hospitalization over the previous 5 years. All patients had a normal or subnormal glomerular filtration rate (>70 ml/min/1.73 m^2^). White cell counts were normal, as were total lymphocyte counts (median: 1680 lymphocytes/mm^3^; range: 1540–1970).

**Table 1:** Demographic characteristics of the 51 diabetic patients.

		N (%) or Median value	Range
Age (years)		55.6	(22–72)
Gender; M/F		33/18 (65/35)	-
Ethnic origin			
	Africa	10 (20)	
	North Africa	14 (27)	
	Indian Continent	4 (8)	
	Western Europe	23 (45)	
Time from diagnosis (years)		8.6	(4.1–39)
Body Mass Index (kg/m^2^)		29.2	(18.9–44.0)
Current treatments			
	Insulin	23 (45)	-
	Oral anti-diabetic drugs	39 (77)	-
	Statins	30 (59)	-
HbA1c level (%)		8.8	(6.0–16.6)
Lipid profile			
	Cholesterolemia (mmol/L)	4.59	(3.60–7.68)
	HDL (mmol/L)	1.32	(0.60–2.21)
	LDL (mmol/L)	2.64	(0.91–3.70)
	Triglyceridemia (mmol/L)	1.59	(0.52–4.05)
MDRD (ml/min/1.73 m^2^)		89.4	(72.9–125.7)
Microalbuminuria (mg/day)		34.6	(6–336)
Prior infection(s)		24 (47)	–
Lymphocyte counts (/mm^3^)		1680	(1540–1970)

### Altered NK Cell Phenotype in Type 2 Diabetic Patients

**Figure 1:**
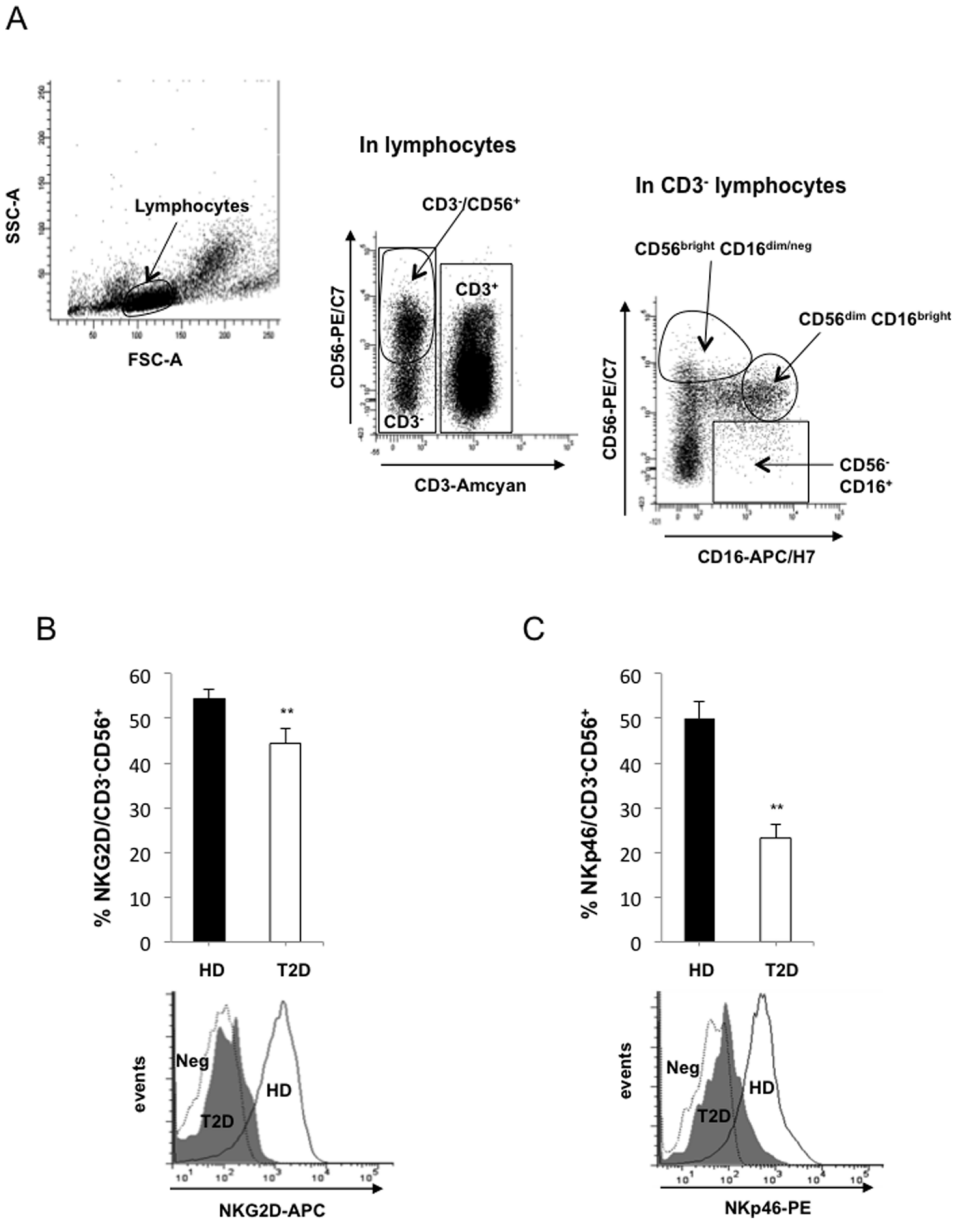
Decreased expression of NKG2D and NKp46 on NK cells from type 2 diabetes patients. (A) Representative gating on CD3^−/^CD56^+^, CD56^bright^ CD16^dim/ne^, CD56^dim^ CD16^bright^ and CD56^−^ CD16^+^ cells. (B) NKG2D expression on NK cells from 25 healthy donors (HD) and 35 type 2 diabetic patients (T2D) as assessed by flow cytometry. One representative flow cytometry experiment is shown. (C) NKp46 expression quantified by FACS (n = 25); a representative analysis is shown. (**: P<0.01).

To determine whether certain immune cell populations were altered in T2D patients, we first extensively immunophenotyped peripheral blood lymphocytes. No difference was observed between T2D patients and control patients for total NK cells, CD4-positive cells, CD8-positive T cells and memory T cell (CD45RO+) frequencies (data not shown). Because we suspected a role for NK cells in the T2D phenotype, we next focused on specific NK cell receptors (Table 2): NKG2D, NKG2C, NKp30, NKp44, NKp46 - all of which are activating receptors, and NKG2A and Killer cell Immunoglobulin-like Receptors (KIRs) - which include inhibitory receptors [13]. NKG2D is expressed on both CD8^+^ T cells and on NK cells. In samples from diabetic patients compared to samples from healthy blood donors, the frequency of NKG2D-expression on CD8^+^ T cells was not modified; however it was significantly lower on NK cells from diabetic patients when compared to healthy blood donors (44 *vs*. 56%, P<0.01), as shown in Figure 1A. The frequency of NKp46 positive cells was also significantly decreased in diabetic patients (26 *vs.* 50%, P<0.01; Figure 1B). Among the inhibitory receptors, NKG2A frequency was decreased in T2D patients whereas KIRs expression was unchanged (Table 2).

**Table 2:** T cell and NK cell immunophenotypes in patients with type 2 diabetes and healthy donors.

		Healthy donors n = 25a,b	T2D patients n = 35a,b	
		% (range)	% (range)	P-value
T Cells				
	CD3+/Lymphocytes	71 (68–80) d	70 (64–77)	NS
	CD4+/CD3+	52 (47–59)	55 (43–62)	NS
	CD8+/CD3+	37 (32–41)	37 (20–48)	NS
	NKG2D+/CD8+	81 (74–85)	80 (68–91)	NS
				
Total NK Cells/Lymphocytes		12 (9–13)	11 (9–15)	NS
Total NK cells				
	NKG2D+	55 (48–59)	44 (31–54)	0.008
	NKp30+	4 (3–10)	3 (1–8)	NS
	NKp44+	2 (1–4)	2 (1–4)	NS
	NKp46+	50 (30–59)	23 (21–43)	0.002
	NKG2C+	9 (3–12)	13 (5–24)	NS
	NKG2A+	30 (23–47)	22 (15–33)	0.03
	CD158a/h+	13 (8–16)	12 (6–27)	NS
	CD158b+	16 (12–22)	13 (11–23)	NS
	CD158e+	9 (4–12)	10 (4–13)	NS
	CD158i+	5 (1–16)	3 (1–9)	NS
				
CD56^bright^ NKCells/Lymphocytes		2 (1–3)	2 (1–3)	NS
CD56^bright^ NK Cells				
	NKG2D+/CD56^bright^	62 (45–76)	58 (45–79)	NS
	NKp30+/CD56^bright^	3 (2–20)	10 (1–31)	NS
	NKp44+/CD56^bright^	12 (4–30)	13 (2–25)	NS
	NKp46+/CD56^bright^	79 (58–88)	81 (65–93)	NS
	NKG2C+/CD56^bright^	12 (6–22)	19 (11–49)	0.03
	NKG2A+/CD56^bright^	48 (33–68)	50 (24–62)	NS
	CD158a/h+/CD56^bright^	5 (3–10)	11 (2–28)	NS
	CD158b+/CD56^bright^	4 (1–8)	6 (4–13)	0.05
	CD158e+/CD56^bright^	1 (0–4)	2 (1–4)	NS
	CD158i+/CD56^bright^	10 (2–32)	5 (2–30)	NS
CD56^dim^ NK Cells/Lymphocytes		10 (6–11)	9 (5–11)	NS
CD56^dim^ NK Cells				
	NKG2D+/CD56^dim^	75 (67–86)	71 (49–83)	NS
	NKp30+/CD56^dim^	4 (2–13)	6 (2–14)	NS
	NKp44+/CD56^dim^	1 (0–1)	1 (0–3)	NS
	NKp46+/CD56^dim^	64 (52–73)	56 (38–64)	NS
	NKG2C+/CD56^dim^	10 (4–16)	22 (10–38)	0.01
	NKG2A+/CD56^dim^	49 (35–66)	31 (20–52)	0.04
	CD158a/h+/CD56^dim^	23 (11–28	19 (12–37)	NS
	CD158b+/CD56^dim^	33 (29–34)	33 (26–43)	NS
	CD158e+/CD56^dim^	20 (8–30)	17 (9–22)	NS
	CD158i+/CD56^dim^	3 (1–47)	3 (1–22)	NS
Ratio CD56^dim^/CD56^bright^ NK cells		5 (2–9)	4 (2–8)	NS
CD3−/CD56−/CD16+Cells/Lymphocytes		5 (3–7)	5 (2–8)	
CD3−/CD56−/CD16+ cells				
	NKG2D+	33 (20–47)	23 (9–41)	NS
	NKp30+	5 (2–12)	3 (1–6)	NS
	NKp44+	1 (0–2)	2 (1–3)	NS
	NKp46+	24 (15–37)	13 (7–28)	0.01
	NKG2C+	5 (3–9)	13 (6–25)	0.005
	NKG2A+	18 (12–23)	26 (18–30)	NS
	CD158a/h+	8 (5–11)	6 (4–12)	NS
	CD158b+	10 (6–18)	17 (5–24)	NS
	CD158e+	9 (4–12)	9 (3–16)	NS
	CD158i+	4 (1–12)	1 (0–7)	NS

a NKp30, NKp44 and NKp46 were analysed in 25 T2D patients and 25 healthy donors.

b CD 158 a/h, b, e and i were analysed in 19 T2D patients and 19 healthy donors.

c P-value T2D patients versus healthy donors, NS: P>0.05.

d Median (Interquartile range).

According to their phenotypes and functional capacities, two NK cell subpopulations can be defined based on CD56 and CD16 expression [28], as follows: CD56^bright^CD16^neg/low^ and CD56^dim^CD16-positive (hereafter referred to as CD56^bright^ and CD56^dim^ NK cells, respectively). CD56^bright^ NK cells tend to produce more cytokines, while CD56^dim^ cells, the major subset at the periphery, are tend to display cytolytic activity [28]. No imbalance between CD56^bright^ and CD56^dim^ NK subpopulations was detected in diabetic patients (Table 2). A third NK cell subset, described as “anergic” (CD56-negative/CD16 positive) [29], was not modified in T2D patients, as shown in Table 2.

One of the ligands of NKG2D is MICA, which exists in a soluble and a cell-bound form. The lower frequency of NKG2D-positive NK cells in T2D patients could be linked to increased soluble MICA production. To test this, we assayed serum MICA concentrations. No difference between diabetic and control groups was found (81+/−17 pg/mL *vs.* 108+/−15 pg/mL, ns; data not shown).

### Correlation between Uncontrolled Diabetes and Reduced NKG2D Expression

**Figure 2:**
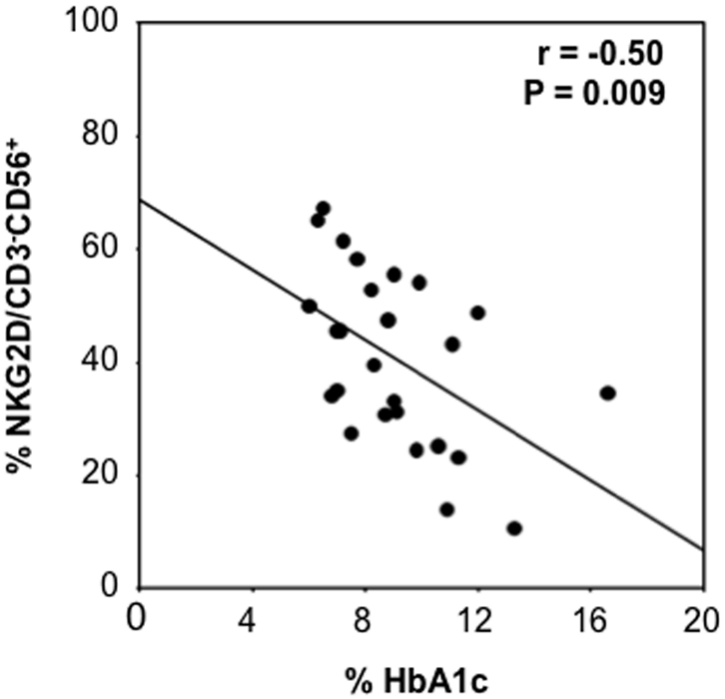
Correlation between plasma HbA1c levels and NKG2D expression on NK cells assesed by Spearman’s correlation. A significant inverse correlation (n = 32, r = −0.5; P = 0.009) is revealed between the main marker of diabetic metabolic control and NKG2D expression on NK cells.

Uncontrolled diabetes, which can be evaluated by HbA1c expression, may contribute to effects on the NK cell population. We therefore compared HbA1c and NK cell receptor expression. Patients with uncontrolled diabetes had the lowest levels of NKG2D expression and a significant inverse correlation was observed between NKG2D- expressing NK cells from diabetic patients and HbA1c levels (r = −0.50; P = 0.009) (Figure 2). Such a correlation was also present when NKG2D MFI quantification was used (r = −0.35; P = 0.04). No significant correlation was observed between NKp46 expression and HbA1c levels.

### Altered NK Cell Function in Type 2 Diabetic Patients

**Figure 3:**
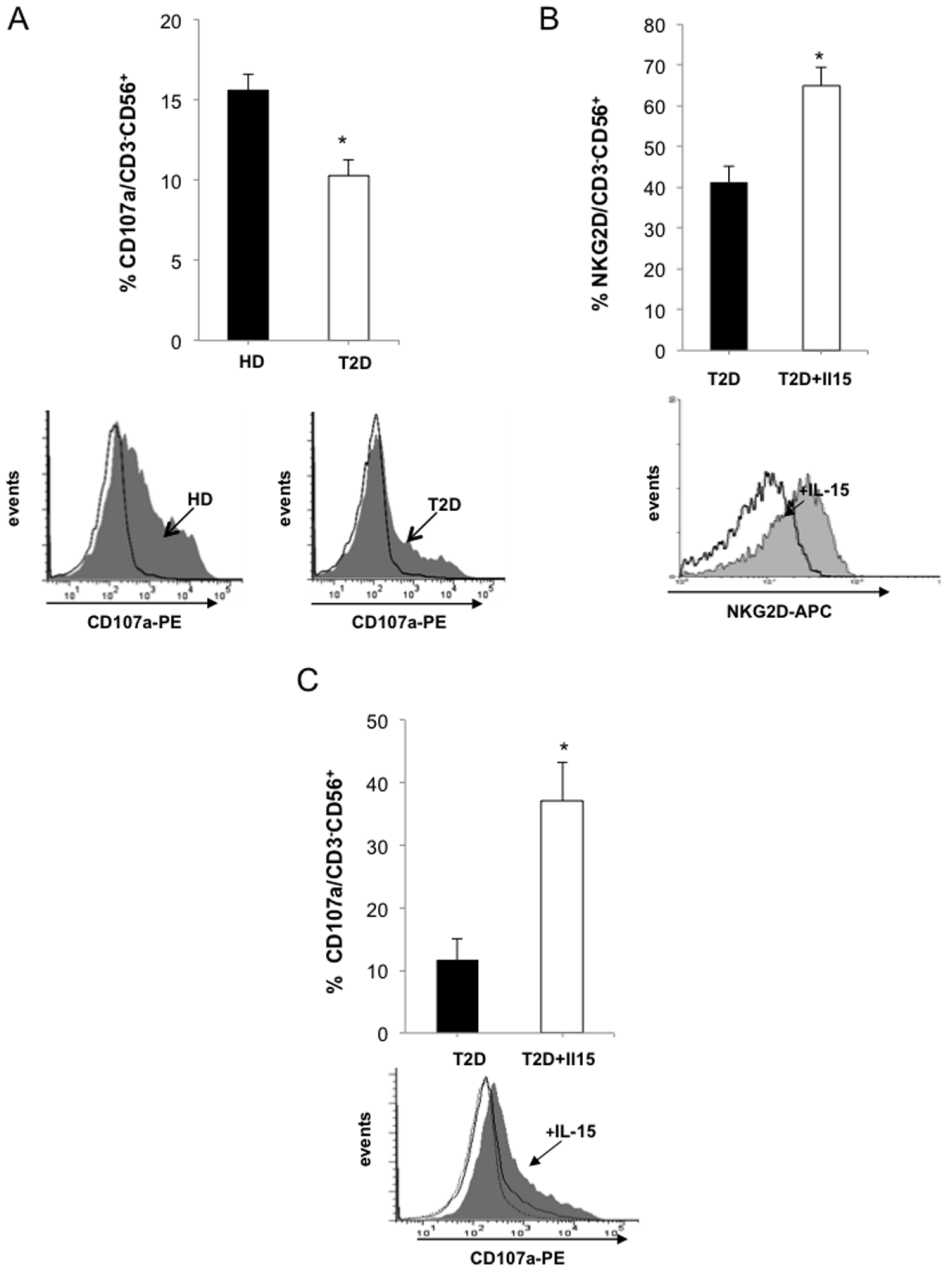
Decreased functional properties of diabetic NK cells and effects of IL-15. (A) CD107a degranulation assay using PBMCs from diabetic (n = 18) or control subjects (n = 16) challenged with K562 cells. Representative flow cytometry experiments are shown. (B) Overnight incubation in the presence of IL-15 (10 ng/mL) significantly increases NKG2D expression on NK cells from T2D patients (n = 7), as assessed by flow cytometry. A representative flow cytometry experiment is shown. (C) Stimulation with IL-15 (10 ng/mL) restores CD107a degranulation for PBMCs from diabetic patients (n = 7) challenged with K562 target cells. A representative flow cytometry experiment is shown *: P<0.05.

To evaluate the functional properties of diabetic NK cells, we tested their ability to degranulate in the presence of target cells. Lysosomal-associated membrane protein-1 (LAMP-1 or CD107a) has been described as a marker of NK cell degranulation following stimulation [30]. Degranulation assays, measuring CD107a translocation to the cell surface, were used to compare performance of NK cells from diabetic patients and healthy controls when faced with K562, a commonly used NK-cell target. In these assays, cells from diabetic patients had a significantly decreased degranulation ability compared to NK cells from healthy donors (10.3+/−1.7% *vs.* 15.8+/−1.4%, P<0.05; Figure 3A). Spontaneous NK cell degranulation was not different between HD and T2D patients (5.1%+/−0.8 vs 4.3% +/−0.7 respectively; ns). Because IL-15 is a key cytokine for NK cell differentiation and maturation [31] and is known to increase NKG2D surface expression [32], we assessed how NK cells freshly isolated from diabetic patients responded to IL-15. Adding IL-15 (10 ng/mL) to culture medium reversed NK cell abnormalities (Figure 3), significantly increasing both NKG2D expression (41.1+/−4% *vs.* 64.9+/−4.6% P<0.05; Figure 3B) and degranulation capacity (11.7% +/−3.3 *vs.* 37.1% +/−6.2, P<0.05; Figure 3C). Circulating IL-15 levels were measured in 30 T2D patients and 20 healthy blood donors, and were found to be similar in both groups (1.29+/−0.23 pg/mL and 0.61 pg/mL+/−0.16 pg/mL respectively, ns).

### NKG2D and NKp46 Transcription in Diabetic Patients

**Figure 4:**
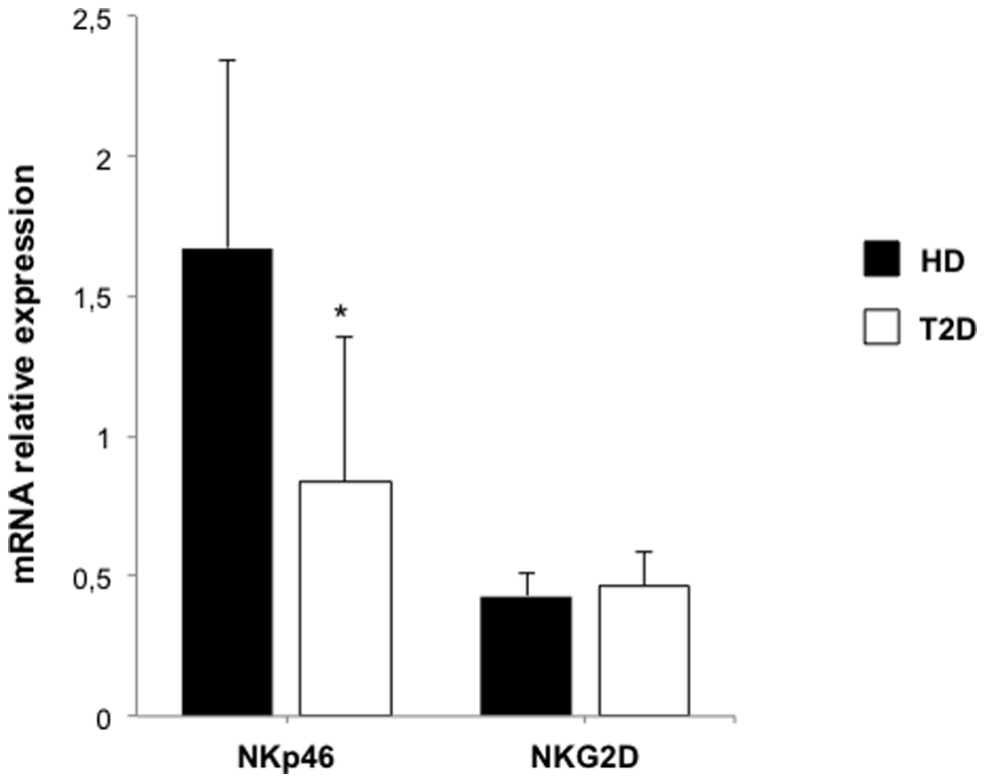
Decreased NKp46 mRNA expression in NK cells from type 2 diabetic patients. Quantitative RT-PCR was used to assess expression of NKp46 and NKG2D mRNAs in NK cells from healthy donors (n = 7) and diabetic patients (n = 7). Transcript levels are presented as mean±SEM. Only NKp46 mRNA is down-regulated. *:P<0.05.

To further characterize the mechanisms resulting in reduced expression of NKG2D and NKp46 in T2D patients, NKG2D and NKp46 mRNAs were quantified by quantitative real-time PCR in NK cells from both healthy controls and diabetic patients. NKp46 mRNA expression was significantly decreased in diabetic patients compared to healthy donors (P<0.05; Figure 4). In contrast, the decreased expression of NKG2D does not appear to be transcriptionally-mediated, since NKG2D mRNA expression was in the same range in healthy donors and diabetic patients (Figure 4)**.** Interestingly, NKG2C mRNA expression was also unaffected in samples from diabetic patients (data not shown).

### ER Stress Contribution to NKG2D Down-modulation in vitro

**Figure 5:**
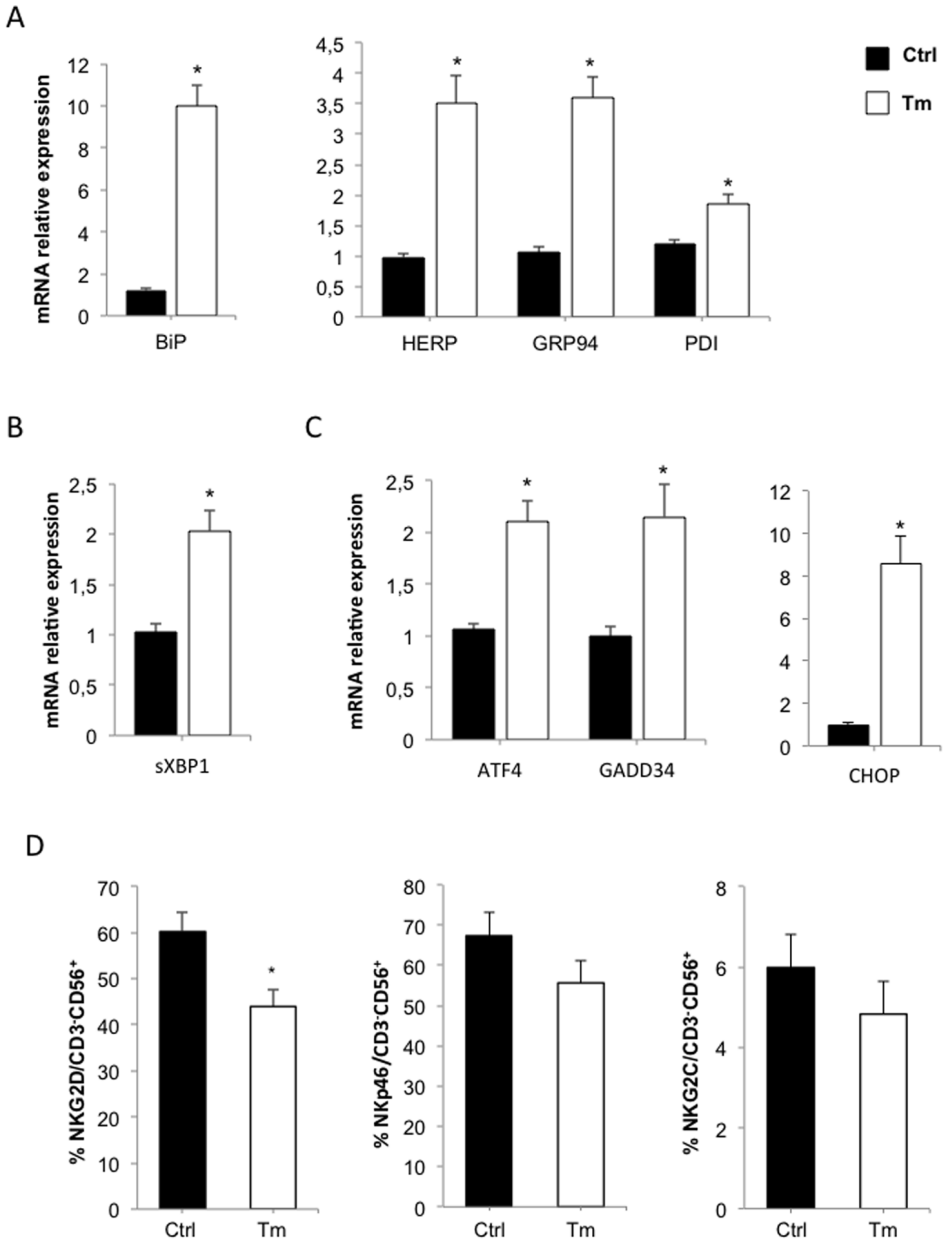
Tunicamycin induces ER stress and UPR activation, and decreases NKG2D expression in normal PBMCs ***in vitro*** **.** PBMCs from healthy donors were incubated in the absence (Ctrl) or in the presence (Tm) of 1.25 µg/mL tunicamycin for 6 hours. (A) ER stress markers *BiP*, *HERP*, *GRP94* and *PDI*, (B) IRE1α pathway marker *spliced XBP1* (s*XBP1*) and (C) PERK pathway markers *ATF4*, *GADD34* and *CHOP* mRNA amounts were determined by quantitative RT-PCR in normal PBMCs. Transcript levels are presented as mean±SEM (n = 6). *:P<0.05. (D) NKG2D, NKp46 and NKG2C expressions were quantified by flow cytometry (n = 12). *:P<0.05.

Since NKG2D mRNA was not under-expressed in NK cells from T2D patients, we hypothesized that ER stress could be involved in reducing cell-surface expression of NKG2D. To test this, we first incubated normal PBMCs in the presence or absence of 1.25 µg/mL tunicamycin, which is known to induce the UPR response, and thus ER stress. ER stress marker mRNAs were quantified by real-time PCR, and revealed overall ER stress to be induced (Figure 5A): BiP, HERP, GRP94 and PDI mRNAs were all significantly increased in tunicamycin-treated samples (with fold increase of 9.43, 3.74, 3.73 and 1.89, respectively). Tunicamycin activates UPR signalling both through the IRE1α pathway (spliced XBP1mRNA increases 2-fold; Figure 5B), and through the PERK pathway (Figure 5C), as indicated by increased expression of ATF4, GADD34 and CHOP mRNAs (Fold-increases of 2.14, 2.12 and 8.19 respectively). Tunicamycin stimulation also decreased NKD2D expression on healthy NK cells: 41.8% +/−3.7 *vs.* 64.3% +/−3, P<0.05 (Figure 5D). NKp46 expression (55.6% +/−5.5 *vs.* 67.3% +/−6.0, ns) and NKG2C expression (4.8% +/−0.8 *vs.* 6.0% +/−0.8 ns) were not significantly affected by tunicamycin treatment (Figure 5D). Tunicamycin did not change NKG2D cell-surface expression on CD3^+^ T cells (data not shown). This suggests that ER stress may be involved in the reduced NKD2D levels observed for NK cells from T2D patients.

### ER Stress and NKG2D Down-regulation in vivo

**Figure 6:**
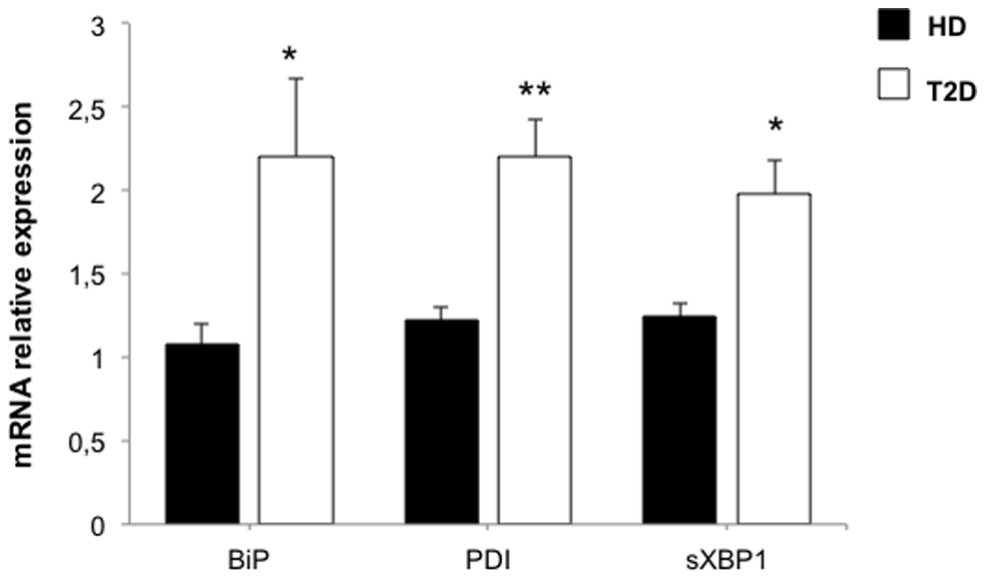
Assessing ER stress and UPR activation in NK cells from diabetic patients. mRNA levels for *BiP*, *PDI* and s*XBP1* were quantified by quantitative RT-PCR in NK cells from healthy donors (n = 17) and type 2 diabetic patients (n = 18). Transcript levels are presented as mean±SEM. *:P<0.05; **: P<0.01.

To confirm the relevance of these in vitro data, NK cells from T2D patients were tested for ER stress markers (Figure 6). In NK cells from T2D patients, ER stress was found to be stimulated with significant increases in BiP and PDI mRNAs (2.2-fold increase for both mRNAs, P<0.05 and P<0.01 respectively). The IRE1α pathway of the UPR was also activated as shown by a 1.98 fold-increase in SXBP1 mRNA in diabetic patients (P<0.05). These data suggest that ER stress and UPR could be both involved in the immune cell phenotype observed in T2D patients.

## Discussion

Epidemiological data shows that T2D is a rapidly growing public health problem [33]. Diabetes is a well-known risk factor for infectious disease, but is also considered as a risk factor for neoplasia [1], [2]. Indeed, a recent cohort study including 28,900 patients found T2D patients to be at increased risk of developing endometrial, renal, gall bladder, liver and pancreatic cancers [2].

NK cells play a crucial role in controlling infections and tumors, but little data is available on this cell subset in T2D patients. In this study, we report that T2D patients have an abnormal NK cell phenotype with a significant decrease in frequency of both NKp46- and NKG2D-positive NK cells. NKp46 is a specific NK receptor which recognizes various influenza hemagglutinins and as yet unknown tumor ligands [34]. It plays a significant role in the eradication of tumor cells in vivo [35]. NKG2D is also an activating receptor present on the plasma membrane of both NK cells and CD8-positive lymphocytes. The best known ligand of NKG2D is the HLA class I-related molecule MICA, which can be found surface-expressed on infected or tumor cells, but also has a circulating soluble form [14]. This soluble form has been shown to modulate NKG2D, expression [36]. We therefore measured soluble MICA in the serum of diabetic patients. Circulating MICA levels in T2D serum were similar to the levels detected in serum from healthy donors. Thus, we conclude that the soluble form of MICA is not responsible for the decreased NKG2D expression observed in T2D patients.

In addition to decreased expression of both NKG2D and NKp46, functional defects were observed for the NK cell population in T2D patients, which showed reduced degranulation. Our results therefore show altered NK cell phenotype and function in T2D patients compared to age-matched healthy blood donors. These observations could partially explain why T2D patients are more at risk for both infection and cancer.

The litterature presents contradictory results with regard to NKG2D expression on cells from patients with diabetes. A decreased frequency of both NKp46 and NKG2D receptors was previously described in a cohort of type 1 diabetic patients [37]. Rodacki et al. [37] reported a direct relationship between altered NK cell phenotype and the duration of type 1 diabetes, and suggest that NK cell perturbations could be linked to the underlying autoimmune disease. In addition, using NKp46-deficient mice, Gur et al. [38] clearly showed that NK cells, particularly through triggering of their NKp46 activating receptor, play a key role in initiating type 1 diabetes. Divergent results were obtained from Akesson et al. who found an increased NKG2D frequency in NK cells from type 2 diabetic patients [39]. In this study, all the included patients had in fact Latent Autoimmune Diabetes in Adults (LADA) with positive anti-GAD autoantibodies, and therefore do not correspond to the “classical” type 2 diabetes phenotype of our cohort.

In the present study, we propose that sustained hyperglycemia itself, which is the hallmark of diabetes, could be directly or indirectly responsible for some of the NK cell defects observed in T2D patients. In corroboration of this hypothesis, blood HbA1c levels correlated strongly with reduced NKG2D expression in T2D patients. HbA1c is considered to be a good marker of how well diabetes was controlled over the three months preceding its quantification. The correlation between this marker and the frequency of NKG2D-positive cells suggests that the NK cell alterations observed may be directly related to high glucose levels. As the incidence of various infections is associated with episodes of severe hyperglycemia, our results offer a possible mechanistic explanation for this association.

In our study, NKp46 down-regulation appears to be transcriptionally-controlled, whereas NKG2D expression is controlled post-transcriptionaly in T2D patients. Since recent evidence suggests that hyperglycemia-induced cell dysfunction may occur as a result of increased ER stress [21], [25], we tested the hypothesis that ER stress could affect NK cell functions by modulating NKG2D surface expression. We studied the three major sensors involved in the UPR response [40], [41]. The first, PERK, is involved in attenuating translation, to reduce the accumulation of unfolded proteins in the ER. In normal NK cells treated with tunicamycin, the PERK pathway is activated as demonstrated by up-regulation of ATF4 and GADD34 mRNAs. The second UPR sensor, IRE1 activation, leads to XBP1 mRNA splicing. XBP1 mRNA codes for a protein which translocates to the nucleus where it induces several ER stress response genes including BiP and PDI. XBP1 mRNA is spliced in NK cells, both in vitro, as shown in the experiments with tunicamycin, and in vivo, in NK cells from T2D patients. The third UPR sensor, ATF6, direcly influences CHOP expression through a pathway potentially leading to apoptosis. No expression of CHOP was observed in NK cells from T2D patients, probably because the level of ER stress is not severe enough. Alternatively, CHOP induction may be delayed compared to other protective factors, in line with its expression in LPS-treated macrophages [42]. It is not currently known how endoplasmic reticulum stress affects NK cells. We show that the ER stress inducer tunicamycin had very little effect on NKp46 and NKG2C expression while it significantly affected NKG2D cell surface expression. Therefore, we assume that ER stress may be one novel mechanism among others involved in the complex regulation of NKG2D cell surface expression.

Having observed these NK cell defects, we wished to determine whether they were reversible. To address this, diabetic NK cells were incubated in the presence of IL-15, a key cytokine for NK cell maturation, which is known to increase NKG2D surface expression [31]. In the presence of IL-15, NK cell degranulation was fully restored, indicating that IL-15 retains its capacity to enhance NK cell activity, even when these cells have been exposed to sustained hyperglycemia. Wether IL-15 by itself may participate in ER stress reversal remains an open issue for future studies.

In conclusion, our results demonstrate that specific NK cell defects could be directly linked to the diabetic milieu. These defects could in turn be linked to the secondary pathologies associated with T2D, infection and neoplasia. This study also highlights the role of metabolic disorders on NK cell dysfunction, especially on the NKG2D/ligand axis. This is a novel field of investigations with a wide medical impact. Pertinently, Xia et al. recently documented the role of NKG2D in atherosclerotic plaque formation [43]. Finally, the results presented here suggest for the first time that hyperglycemia-mediated ER stress plays a role in the NK cell dysfunction accompanying diabetes. Further study will be required to establish the kinetics of this relationship, and to determine the effects of anti-diabetic treatments on the expression of ER stress proteins in the course of diabetes. Studies will also be required with appropriate glycemic controls to analyze whether the severity and duration of hyperglycemia affects the expression of ER stress proteins and their consequences on the immune status of diabetic patients.

## Supporting Information

Table S1 **Antibodies and colors used for FACS experiments.**
(DOC)

Table S2 **Primer sequences used for RT-PCR analysis.**
(DOC)
