# Correction: Natural Killer Cell Function, an Important Target for Infection and Tumor Protection, Is Impaired in Type 2 Diabetes

**DOI:** 10.1371/annotation/c6fd65ca-7ab4-4407-ac23-2b9144073dce

**Published:** 2014-01-02

**Authors:** Jeannig Berrou, Sophie Fougeray, Marion Venot, Victor Chardiny, Jean-François Gautier, Nicolas Dulphy, Antoine Toubert, Marie-Noëlle Peraldi

Dr. Philippe Haas was not included in the author byline. He should be listed as the fifth author, and his affiliation is: Institut National de la Santé et de la Recherche Médicale (INSERM), UMR 940, F-75010, Paris, France; Assistance Publique-Hôpitaux de Paris (AP-HP), Hôpital Saint-Louis Centre d’Investigations Biomédicales "H-O-G", Paris, France; Université Paris 7-Denis Diderot, Sorbonne Paris Cité, Paris, France; Université Paris Diderot, Sorbonne Paris Cité, Institut Universitaire d'Hématologie, F-75010, Paris, France. The contributions of this authors are as follows: Analyzed the data.

The correct citation is: Berrou J, Fougeray S, Venot M, Chardiny V, Haas P, et al. (2013) Natural Killer Cell Function, an Important Target for Infection and Tumor Protection, Is Impaired in Type 2 Diabetes. PLoS ONE 8(4): e62418. doi:10.1371/journal.pone.0062418 

