# Supplementary material for: Natural Killer Cell Function, an Important Target for Infection and Tumor Protection, Is Impaired in Type 2 Diabetes
Source: PLoS One. 2013 Apr 25;8(4):e62418. doi: 10.1371/journal.pone.0062418 (PMC3636194; doi:10.1371/journal.pone.0062418)
Supplement: Table S1 — Antibodies and colors used for FACS experiments. (DOC) [file pone.0062418.s001.doc]

**Supplemental table S1.** Antibodies and colors used for FACS experiments.

| Tube | FITC | PE | PerCP | APC | Streptavidin PE-Alexa Fluor 750 | APC-H7 | Amcyan | Pacific Blue |
| --- | --- | --- | --- | --- | --- | --- | --- | --- |
| 1 |  | NKp46 | CD8 | NKG2D | Biotin anti-CD56 | CD16 | CD3 | CD4 |
| 2 |  | NKp44 |  |  | Biotin anti-CD56 | CD16 | CD3 |  |
| 3 |  | NKp30 |  |  | Biotin anti-CD56 | CD16 | CD3 |  |
| 4 |  | NKG2C | CD8 | NKG2A | Biotin anti-CD56 | CD16 | CD3 | CD4 |
| 5 | CD158b | CD158a/h |  | CD158e | Biotin anti-CD56 | CD16 | CD3 |  |
| 6 |  | CD158i |  |  | Biotin anti-CD56 | CD16 | CD3 |  |
