# Supplementary material for: Natural Killer Cell Function, an Important Target for Infection and Tumor Protection, Is Impaired in Type 2 Diabetes
Source: PLoS One. 2013 Apr 25;8(4):e62418. doi: 10.1371/journal.pone.0062418 (PMC3636194; doi:10.1371/journal.pone.0062418)
Supplement: Table S2 — Primer sequences used for RT-PCR analysis. (DOC) [file pone.0062418.s002.doc]

**Supplemental table S2.** Primer sequences used for RT-PCR analysis

| Gene name | PCR product size* | Primer sequences | Final concentration |
| --- | --- | --- | --- |
| ATF4 | 226 | F 5’-TCAAACCTCATGGGTTCTCC-3’  R 5’-GTGTCATCCAACGTGGTCAG-3’ | 300 nM |
| BiP/GRP78 | 200 | F 5’-GGTGAAAGACCCCTGACAAA-3’  R 5’-GTCAGGCGATTCTGGTCATT-3’ | 300 nM |
| CHOP | 170 | F 5’-TGGAAGCCTGGTATGAGGAC-3’  R 5’-TGTGACCTCTGCTGGTTCTG-3’ | 300 nM |
| GADD34 | 171 | F 5’-AGGAGGCTGAAGACAGTGGA-3’  R 5’-GGCCATCTGCAAATTGACTT-3’ | 300 nM |
| GAPDH | 109 | F 5’-GCCATCAATGACCCCTTCATT-3’  R 5’-TTGACGGTGCCATGGAATTT-3’ | 50 nM |
| GRP94 | 285 | F 5’-AGTACGGATGGTCTGGCAAC-3’  R 5’-TGAGGCGAAGCATTCTTTCT-3’ | 300 nM |
| HERP | 178 | F 5’-TGAAACTGAAGACCCCAACC-3’  R 5’-CCTCCAACAGCTACAGCACA-3’ | 300 nM |
| NKG2C | 190 | F 5’-AGTCATCATCCATGGGTGACAAT-3’  R 5’-CTGATGCACTGTAAACGCAAATG-3’ | 400 nM |
| NKG2D | 294 | F 5’-GGTATGAGAGCCAGGCTTCTTGT-3’  R 5’-CTTTACACAGTCCTTTGCATGCA-3’ | 100 nM |
| NKp46 | 179 | F 5’-AGAATCTCCTTCGGATGGGC-3’  R 5’-CAGCTTTCAGATCCACCCATG-3’ | 400 nM |
| PDI | 145 | F 5’-GCTGTCATCGGCTTCTTC-3’  R 5’-CAACCCCATCTTTGTCG-3’ | 300 nM |
| RPL13A | 124 | F 5’-CCTGGAGGAGAAGAGGAAAGAGA-3’  R 5’-GAGGACCTCTGTGTATTTGTCAA-3’ | 300 nM |
| sXBP1 | 117 | F 5’-GCAGGTGCAGGCCCAGTTGT-3’  R 5’-TGGGTCCAAGTTGTCCAGAATGC-3’ | 300 nM |

* nucleotides
